# Comprehensive Genome-Wide Transcriptomic Analysis of Immature Articular Cartilage following Ischemic Osteonecrosis of the Femoral Head in Piglets

**DOI:** 10.1371/journal.pone.0153174

**Published:** 2016-04-05

**Authors:** Naga Suresh Adapala, Harry K. W. Kim

**Affiliations:** 1 Center for Excellence in Hip Disorders, Texas Scottish Rite Hospital for Children, Dallas, Texas, 75219, United States of America; 2 Department of Orthopedic Surgery, University of Texas Southwestern Medical Center, Dallas, Texas, 75390–8883, United States of America; University of Massachusetts Medical, UNITED STATES

## Abstract

**Objective:**

Ischemic osteonecrosis of the femoral head (ONFH) in piglets results in an ischemic injury to the immature articular cartilage. The molecular changes in the articular cartilage in response to ONFH have not been investigated using a transcriptomic approach. The purpose of this study was to perform a genome-wide transcriptomic analysis to identify genes that are upregulated in the immature articular cartilage following ONFH.

**Methods:**

ONFH was induced in the right femoral head of 6-week old piglets. The unoperated femoral head was used as the normal control. At 24 hours (acute ischemic-hypoxic injury), 2 weeks (avascular necrosis in the femoral head) and 4 weeks (early repair) after surgery (n = 4 piglets/time point), RNA was isolated from the articular cartilage of the femoral head. A microarray analysis was performed using Affymetrix Porcine GeneChip Array. An enrichment analysis and functional clustering of the genes upregulated due to ONFH were performed using DAVID and STRING software, respectively. The increased expression of selected genes was confirmed by a real-time qRTPCR analysis.

**Results:**

Induction of ONFH resulted in the upregulation of 383 genes at 24 hours, 122 genes at 2 weeks and 124 genes at 4 weeks compared to the normal controls. At 24 hours, the genes involved in oxidoreductive, cell-survival, and angiogenic responses were significantly enriched among the upregulated genes. These genes were involved in HIF-1, PI3K-Akt, and MAPK signaling pathways. At 2 weeks, secretory and signaling proteins involved in angiogenic and inflammatory responses, PI3K-Akt and matrix-remodeling pathways were significantly enriched. At 4 weeks, genes that represent inflammatory cytokines and chemokine signaling pathways were significantly enriched. Several index genes (genes that are upregulated at more than one time point following ONFH and are known to be important in various biological processes) including *HIF-1A*, *VEGFA*, *IL-6*, *IL6R*, *IL-8*, *CCL2*, *FGF2*, *TGFB2*, *MMP1*, *MMP3*, *ITGA5*, *FN and Col6A1* were upregulated in the immature articular cartilage following ONFH. A qRTPCR analysis of selected genes confirmed the upregulated expression observed in the microarray analysis.

**Conclusion:**

Immature articular cartilage responds to ONFH by the upregulation of genes involved in hypoxic stress response, angiogenesis, matrix remodeling and inflammation. This study provides novel insights into the multi-faceted role of immature articular cartilage, with inflammation as a key component, following ONFH in piglets.

## Introduction

Legg-Calvé-Perthes disease (LCPD) is a childhood form of ischemic osteonecrosis of the femoral head (ONFH), which can produce a severe flattening deformity of the femoral head [1,2]. This deformity can lead to a debilitating osteoarthritis of the hip joint as early as the third decade. There is no effective medical treatment for LCPD since the pathophysiology of the disease is not well understood [3,4].

In contrast to the mature femoral head in adults, the developing femoral head in children contains a growth cartilage surrounding the secondary center of ossification. The circumferential increase in the size of the secondary ossification center is dependent on the growth cartilage present in the deep layer of the immature articular cartilage just above the secondary ossification center (also known as the epiphyseal cartilage). Following ONFH, a cessation of the growth of the secondary ossification center occurs due to the necrotic damage to the growth cartilage [5,6]. The ischemic damage is followed by increased vascularization of the cartilage, fibrocartilage formation and hypertrophy over time [7–9]. These findings in LCPD patients suggest that the immature articular cartilage undergoes active pathophysiological changes in response to ONFH. In addition, the immature articular cartilage may also contribute to the chronic synovial inflammation in LCPD, which is characterized by a specific and sustained elevation of the pro-inflammatory cytokine interleukin-6 (IL-6) in the synovial fluid [10,11]. Thus, in order to better understand the role of immature articular cartilage following ONFH and to devise novel therapeutic approaches to prevent early onset osteoarthritis following ONFH, the molecular changes in the immature articular cartilage must be assessed.

The piglet model of ischemic osteonecrosis of the femoral head shows pathological features similar to LCPD patients [12–15]. In this model, the placement of a tight ligature around the femoral neck and transection of the ligamentum teres results in a complete disruption of blood supply to the femoral head. This leads to extensive cell death in the deep layer of the immature articular cartilage, but the superficial and proliferative layers remain viable [12,16,17]. The hypoxic stress due to ischemic injury has been demonstrated to significantly upregulate hypoxia-inducible factor-1 (HIF-1) expression in the immature articular cartilage of the piglet model [18] along with increased production of angiogenic and chondrogenic factors, VEGF [19], BMP2 [20] and the chondrocytic transcription factor Sox9 [21]. These studies indicated the possible upregulation of pathways involved in angiogenesis and hypertrophy of the articular cartilage. In pathologic conditions affecting adult articular cartilage like osteoarthritis, the articular cartilage is known to perform regulatory functions including matrix remodeling and inflammation [22].

The purpose of this study was to determine genes that are upregulated in the immature articular cartilage following ONFH and determine the biological processes that are represented by the upregulated genes. To this effect, we performed a microarray analysis to determine the genes that are upregulated at the whole-transcriptome level. The upregulated genes were functionally clustered into enriched gene-groups based on the representation of the genes in the specific biological processes, molecular functions and biological pathways as annotated in the previous literature using DAVID software [23,24] and STRING software [25]. We confirmed the upregulation of key index genes by a real-time quantitative RTPCR analysis.

## Materials and Methods

### Animals

This study was approved by the Institutional Animal Care and Use Committee at the University of Texas Southwestern Medical Center. The Yorkshire piglets (6-week old) used in this study were obtained from Walliser hog farms, Florida and were housed in 19 sq.ft. Cages, 12-hour light cycle, 61–81°F temperature and were fed Purina Nature’s Match Starter Diet (Gray Summit, MO, USA). For analgesia, Carprofen (4mg/kg/PO) was given pre-operatively and post-operatively, Buprenorphine SR (0.3mg/kg/SC) post-operatively. For anesthesia, Telazol (4mg/kg/IM), Isoflurane 1–3% (minimum alveolar concentration), atropine (0.04mg/kg/IM) was used. For 3 days post-operatively, animals were given Penicillin G Procaine (30000U/lb./IM) and Carprofen (4mg/kg/PO). All efforts were made to minimize suffering. At the indicated time points, animals were euthanized by the injection of Phenobarbital Sodium (90mg/kg). A total of 18 piglets were used in the study as indicated below.

### Induction of ischemic osteonecrosis in the femoral head

Ischemic osteonecrosis (ONFH) was induced in the right femoral head by placing a suture ligature tightly around the femoral neck and by transection of the ligamentum teres, as described previously [10,12,15]. Left femoral heads were not operated and served as the control group. For SHAM operation, the femoral neck was exposed but no ligation or transection of the ligamentum teres was performed. The animals in the study were sacrificed at 24 hours, 2 weeks and 4 weeks (n = 4 at each time point) following the surgery. We specifically chose these time points since induction of ischemia causes acute hypoxic stress within 24 hours in the femoral head and the deep layer of the immature articular cartilage based on previous studies using the piglet model [12,16,17]. By 2 weeks, extensive necrosis of the bone and marrow cells in the secondary ossification center and the chondrocytes in the deep layer of the immature articular cartilage are observed. By 4 weeks after the induction of ischemia, early revascularization is observed in the immature articular cartilage and the secondary ossification center [12,14,16,18].

The SHAM operated pigs [n = 2/time point] did not reveal any gross, radiographical or histological changes that would indicate ischemic damage as reported previously [17] (data not shown). Hence, only articular cartilage from normal and osteonecrosis femoral heads was processed for RNA isolation to be used in the microarray analysis.

### RNA isolation from the articular cartilage

Femoral heads from the right (ONFH) and left (normal) hip joints were isolated under sterile conditions and a full-thickness of the immature articular cartilage was harvested using a surgical blade. The articular cartilage was snap frozen using liquid nitrogen. The total RNA from the tissue was collected using a TRIzol reagent (Invitrogen, Carlsbad, CA, USA) according to the manufacturer’s instructions. The total RNA was treated with DNAse I (Ambion, Foster City, CA, USA) and purified by a Qiagen RNeasy Mini Column (Qiagen, Valencia, CA, USA). The RNA concentration was determined by using the Nanodrop ND 1000 spectrophotometer. The integrity of the RNA was determined by using Agilent Bioanalyzer 2100 (Agilent technologies, Santa Carla, CA, USA). The RNA samples used in the study contained an integrity number (RIN) above 7.0, which was considered suitable for a microarray analysis.

### Affymetrix GeneChip Porcine Genome Array

A total of 23,937 probe sets, which interrogate 23,256 transcripts representing 20,201 porcine genes were included in the Affymetrix GeneChip Porcine array (Affymetrix Inc., Santa Carla, CA, USA). The RNA preparation and hybridization were performed according to the manufacturer’s protocol. The gene chips were scanned with the Affymetrix GeneChip Scanner 3000 (Affymetrix, Santa Carla, CA, USA).

### Microarray analysis

The raw data obtained from the Affymetrix GeneChip Scanner in.CEL format was analyzed using Affymetrix Expression Console, which performs the normalization of signal intensity, quality control and performs statistical analysis. For background adjustment, quantile normalization and summarization, a Robust Multichip Analysis (RMA) was used. The data with a bad signal quality was filtered and excluded through a correlation analysis and a Principal Component Analysis (PCA) performed by using the Expression Console. The.CHP files thus generated were analyzed to determine differentially expressed genes (DEGs) between normal and osteonecrosis at 24 hours, 2 weeks and 4 weeks time points. The DEGs were determined by using Affymetrix Transcriptome Analysis Console (TAC), following the software guidelines. For this purpose, t-test, Multiple Testing Corrections and False Discovery Rate Prediction were performed. The genes which showed at least a 2-fold difference between normal and ONFH at different time points, with a p value of less than 0.05, were considered statistically significant and differentially expressed between normal and osteonecrosis (S1 Table). A GeneSpring software (Agilent technologies, Santa Carla, CA, USA) was used to prepare the profile plot to demonstrate changes in the whole transcriptomic expression over 24 hours, 2 weeks and 4 weeks after ONFH surgery. For the enrichment analysis, the following probes were excluded in DAVID [23,24] and STRING [25] analysis: (1) probes for which porcine gene identity was unknown (2) replicate/multiple probes that represent the same gene (3) Probes which were not identified by the DAVID software.

### Gene enrichment analysis and functional clustering of the differentially expressed genes

The data obtained from the Transcription Analysis Console was utilized to generate *gene lists* from the corresponding probe IDs using Affymetrix NetAffyx analysis software. The gene lists were then assessed for the biological significance and a functional clustering.

The specific biological functions, molecular functions and functional pathways that were enriched among the upregulated genes were determined by using the DAVID v6.7 software (The Database for Annotation, Visualization and Integrated Discovery, http://david.abcc.ncifcrf.gov/) [23,24]. The *genes lists* were manually curated and tested for suitability in the DAVID analysis. Briefly, the following factors were considered: (1) whether many of the genes among the upregulated genes are “marker” genes, which play an important role in the interested biological processes, for example inflammation; (2) whether notable portion of the genes are involved in specific rather than generally many biologic processes; (3) more enriched in biology than a random list of genes. For enrichment analysis using DAVID software, the genes in a given list were statistically compared, using standard parameters on DAVID software, to the background list (whole transcriptome) using χ2, Fisher’s exact test, Binomial probability and Hyper-geometric distribution to obtain an Enrichment score, which is the geometric mean of all enrichment p values. The gene lists were statistically assessed by EASE score (Modified Fisher’s Exact test p value 0.05), Bonferroni correction and an FDR (false discovery rate) in order to establish the statistical significance. For all analyses, a medium stringency was used. The biological processes and molecular functions were assessed by DAVID software. The functional clustering of genes upregulated in the articular cartilage into biological pathways (based on KEGG pathways) was performed using a STRING10 (Search Tool for the Retrieval of Interacting Genes/Proteins) database (http://www.string-db.org) [25].

### Quantitative real-time RT-PCR

Reverse transcription of RNA was performed with 1 microgram of RNA using a Superscript First Strand Synthesis System for RT-PCR (Invitrogen, Carlsbad, CA, USA) in a 30μl reaction volume. Using forward and reverse primers for each gene of interest, a BioRad iTaq Universal SYBR Green Supermix (Bio-Rad Laboratories, Hercules, CA, USA) and Applied Biosystems 7500 Real Time PCR System (Thermo Scientific, Waltham, MA, USA) were used to perform the quantitative real-time PCR. Data analysis was performed using the delta-delta CT method. The sequence information for the specific primers used in this study is shown in S2 Table.

### Statistical analysis

The statistical methods used in the processing of the raw data from the Affymetrix GeneChip Scanner were described above. For qRTPCR analysis, ANOVA and Tukey’s multiple comparison were used to determine statistically significant differences between normal and osteonecrosis samples at 24 hours, 2 weeks and 4 weeks. A p value of less than 0.05 was considered as statistically significant.

## Results

### At 24 hours after the induction of ONFH (acute ischemic-hypoxic injury phase), oxidoreductive, cell survival and angiogenesis pathways were upregulated in the immature articular cartilage

ONFH resulted in the differential expression of genes in the immature articular cartilage (S1 Fig). The gene expression profile in the normal cartilage remained similar at 24 hours; 2 weeks and 4 weeks time points. A total of 383 genes, 122 genes and 124 genes were significantly upregulated (>2-fold increase, p<0.05) in the affected cartilage compared to the normal cartilage at 24 hours, 2 weeks, and 4 weeks, respectively (S1 Table).

A functional annotation clustering using DAVID analysis software revealed that acutely (24 hours) after the induction of ischemia, the genes involved in the oxidoreductive stress response and angiogenesis were significantly enriched among the upregulated genes (Table 1 and S1 Table). These processes correspond to protein dimerization (e.g. transcription) and oxidoreductive activity.

**Table 1:** Biological processes, molecular functions and biological pathways upregulated in the immature articular cartilage at 24 hours following ONFH.

**DAVID**	**Enrichment**	**Count**	**P value**	**Bonferroni**	**FDR**
**Annotation category**	**score**				
**GOTERM-BP-FAT**					
Response to endogenous stimulus	5.81	39	3.30E-14	7.50E-11	5.80E-11
Response to organic substrate	5.81	50	1.30E-12	2.90E-09	2.30E-09
Blood vessel development	5	19	4.30E-06	1.90E-02	1.40E-02
**SP_PIR_keyword**					
Oxidoreductase	3.5	29	1.10E-06	4.40E-04	1.50E-03
Angiogenesis	3.45	10	2.50E-06	1.00E-03	3.50E-03
**GOTERM-MF-FAT**					
Protein dimerization	2.93	29	1.20E-05	6.70E-03	1.80E-02
Oxidoreductase activity	2.35	9	7.30E-05	2.00E-02	1.10E-01
Growth factor binding	1.98	10	3.80E-04	1.90E-01	5.60E-01
**STRING-PATHWAYS**	**Count**	**P value**	**Bonferroni**	**FDR**	
HIF-1 signaling pathway	15	3.61E-10	1.04E-07	1.04E-07	
PI3K-Akt signaling pathway	23	3.37E-08	9.67E-06	3.42E-06	
MAPK signaling pathway	16	1.05E-05	3.00E-03	3.75E-04	
Focal adhesion	14	1.47E-05	4.20E-03	4.58E-04	
TNF signaling pathway	10	1.79E-05	5.14E-03	4.67E-04	

**Abbreviations:** ONFH: osteonecrosis of the femoral head; DAVID: The Database for Annotation, Visualization and Integrated Discovery; GOTERM-BP-FAT: gene ontology term-biological process-functional annotation tool; SP_PIR_keyword: SwissPort protein interaction resource keyword, indicates protein categories; GOTERM-MF-FAT: gene ontology term-molecular function-functional annotation tool; STRING-pathways: Search Tool for the Retrieval of Interacting Genes/Proteins; Count: number of genes that are significantly enriched in the indicated annotation category. FDR: false discovery rate. Bonferroni: a statistical test used in the correction for multiple comparisons.

Previous studies have shown that the HIF-1 pathway upregulation was a major response to ONFH [18–21]. In this study, several important genes in the HIF-1 pathway, including transcription factors (*RelA*, *Fos*, *ATF4*), growth factors (*TGFB2*, *FGF2*, *NGF*), survival response elements (*MAP3K5*, *GADD45B*, *AKT3*) and hypoxic response elements (*DDIT3*) were upregulated. Additionally, genes involved cell survival pathways including PI3K-Akt pathway and MAPK pathway were significantly upregulated. In the PI3K-Akt signaling pathway, which is critical for cell survival [26], an upregulation of *ENO2*, *GAPDH*, *CDA and ALDOC* were observed. Genes involved in the MAPK pathway included growth factors (*VEGF*), inflammatory mediators (*IL-6R*) and several matrix related proteins *FN1*, *TNC*, *COL6A1*, *ITGA5 and THBS3*. Several genes in the pathways mentioned above were also commonly involved in focal adhesion and TNF-a signaling pathways.

**Fig 1:**
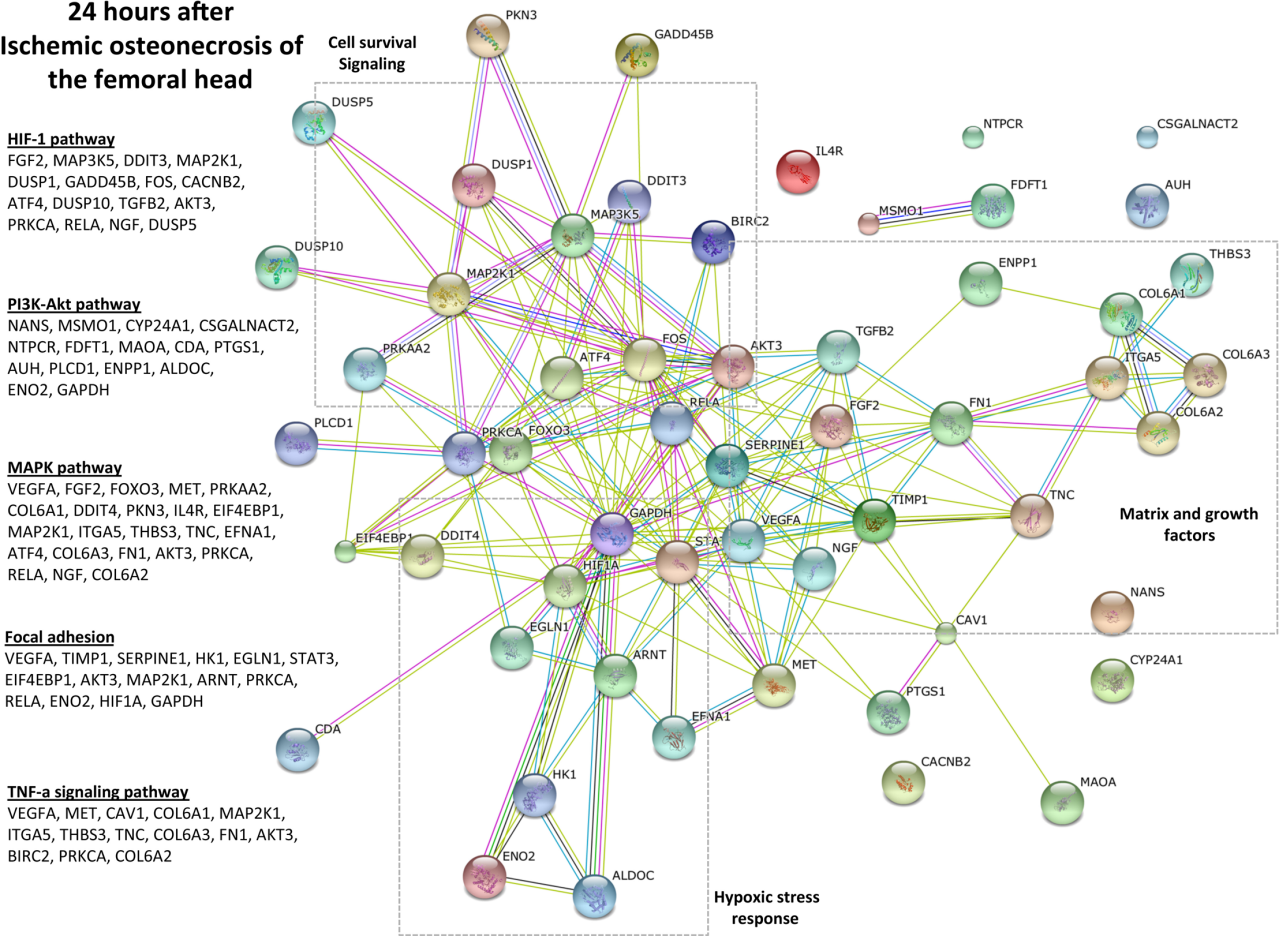
Response to hypoxic stress (HIF-1 pathway) and cell survival signaling pathways (PI3K-Akt, MAPK pathways) are the most significantly enriched functions in the immature articular cartilage at 24 hours following the induction of ONFH. The genes and the specific pathways that were significantly enriched (p<0.05, based on t-tests, Bonferroni, FDR) are listed. The network diagram (STRING10 software) indicates a close relationship of different genes based on the functional roles in biological pathways. The clusters of genes indicating specific biological functions (cell survival and signaling, matrix and growth factors, hypoxic stress response) are indicated using boxes with dashed lines. The nodes indicate individual genes and the connecting lines indicate the relationship. Shorter lines indicate closer relationship. This evidence plot utilizes multiple lines to indicate stronger evidence for the proximity in a functional relationship.

The functional clustering diagram in Fig 1 provided the summary of the gene-groups, with genes more closely related being closer in the network map. These results indicate that the immature articular cartilage responds acutely to ONFH through the upregulation of genes involved in the oxidative response, cell survival and production of growth factors involved in angiogenesis and matrix formation.

### At 2 weeks after the induction of ONFH (avascular necrosis phase), the genes regulating angiogenesis, inflammation and matrix remodeling were upregulated in the immature articular cartilage

At 2 weeks after ONFH, when there is extensive necrosis in the secondary ossification center and marrow region of the femoral head [12,14,16,18]. Similar to 24 hours, genes involved in response to ischemic injury and angiogenesis were significantly enriched at 2 weeks. However, the role of immature articular cartilage in inflammation became evident at 2weeks with upregulation of secreted proteins and signaling components (Table 2 and S2 Table) that correspond to matrix formation, growth factor binding and cell signaling mechanisms.

**Table 2:** Biological processes, molecular functions and biological pathways upregulated in the immature articular cartilage at 2 weeks following ONFH.

**DAVID**	**Enrichment**	**Count**	**P value**	**Bonferroni**	**FDR**
**Annotation category**	**score**				
**GOTERM-BP-FAT**					
Response to wounding	6.69	21	6.90E-10	1.00E-06	1.10E-06
Inflammatory response	6.69	14	4.70E-07	7.00E-04	7.80E-04
Blood vessel development	4.07	10	6.00E-05	2.70E-02	3.10E-02
**SP_PIR_keyword**					
Secreted	14.24	48	2.10E-21	5.30E-19	2.80E-18
Signal	14.24	58	6.40E-17	2.80E-14	1.40E-13
**GOTERM-MF-FAT**					
Extracellular matrix	3.98	15	1.70E-07	1.60E-05	1.90E-04
Growth factor binding	3	7	1.00E-04	3.10E-02	1.40E-01
Signaling molecule	2.34	19	3.80E-06	3.40E-04	4.20E-03
**STRING-PATHWAYS**	**Count**	**P value**	**Bonferroni**	**FDR**	
PI3K-Akt signaling pathway	11	3.10E-06	8.90E-04	4.45E-04	
Cytokine-cytokine receptor interaction	9	1.64E-05	4.71E-03	7.85E-04	
Protein digestion and absorption	6	7.87E-06	2.26E-03	4.84E-04	
Rheumatoid arthritis	6	8.43E-06	2.42E-03	4.84E-04	
ECM-receptor interaction	6	8.43E-06	2.42E-03	4.84E-04	

**Abbreviations:** ONFH: osteonecrosis of the femoral head; DAVID: The Database for Annotation, Visualization and Integrated Discovery; GOTERM-BP-FAT: gene ontology term-biological process-functional annotation tool; SP_PIR_keyword: SwissPort protein interaction resource keyword, indicates protein categories; GOTERM-MF-FAT: gene ontology term-molecular function-functional annotation tool; STRING-pathways: Search Tool for the Retrieval of Interacting Genes/Proteins; Count: number of genes that are significantly enriched in the indicated annotation category. FDR: false discovery rate. Bonferroni: a statistical test used in the correction for multiple comparisons.

**Fig 2:**
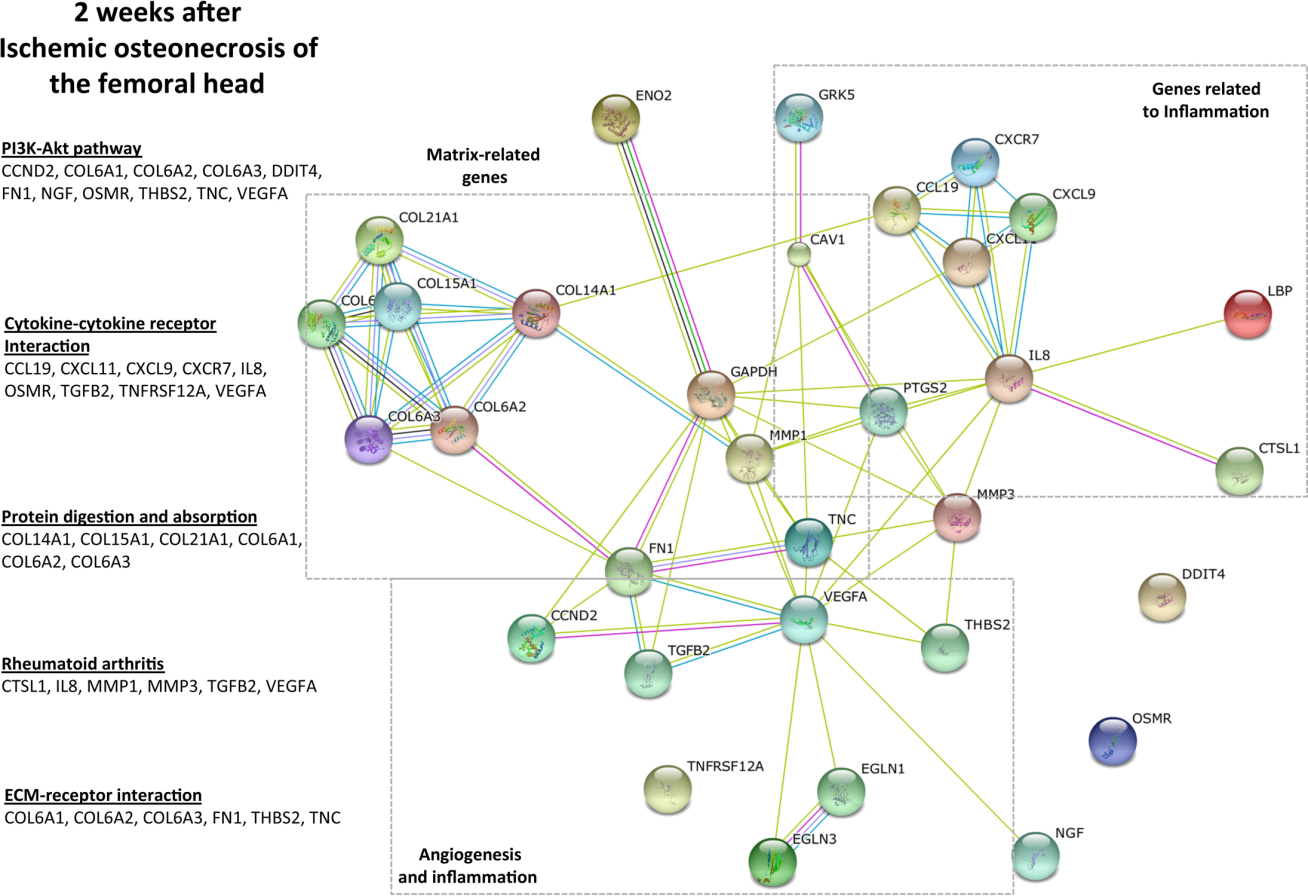
Angiogenesis, matrix remodeling and inflammation are the most significantly enriched functions in the immature articular cartilage at 2 weeks following the induction of ONFH. The genes and the specific pathways that were significantly enriched (p<0.05, based on t-tests, Bonferroni, FDR) are listed. The STRING10 network diagram was used to indicate the closeness of relationship of different genes based on the functional roles in biological pathways. The clusters of genes indicating matrix-related functions (e.g. *FN*, *TNC*, *MMP1*, *MMP3*), angiogenesis (e.g. *VEGF*) and inflammation (e.g. *IL8*, *OSMR*) are shown. The nodes indicate individual genes and the connecting lines indicate the relationship. Shorter lines indicate closer relationship. The evidence plot utilizes multiple lines to indicate stronger evidence for the proximity in a functional relationship.

Similar to 24 hours, PI3K-Akt pathway was significantly upregulated at 2 weeks after ONFH (Fig 2) that included growth factors (*VEGFA*, *NGF*, *THBS2*) and matrix proteins (*FN1*, *TNC*, *Col6A1*). Importantly, several genes involved in the inflammatory response (cytokines/receptors *IL-8*, *OSMR*, *TNFRSF12A*) and several chemokines/receptors (including *CCL2*, *CXCL11*, *CXCR7*) were upregulated. The expression of matrix remodeling enzymes (MMP1, CTSL1) was also upregulated.

**Fig 3:**
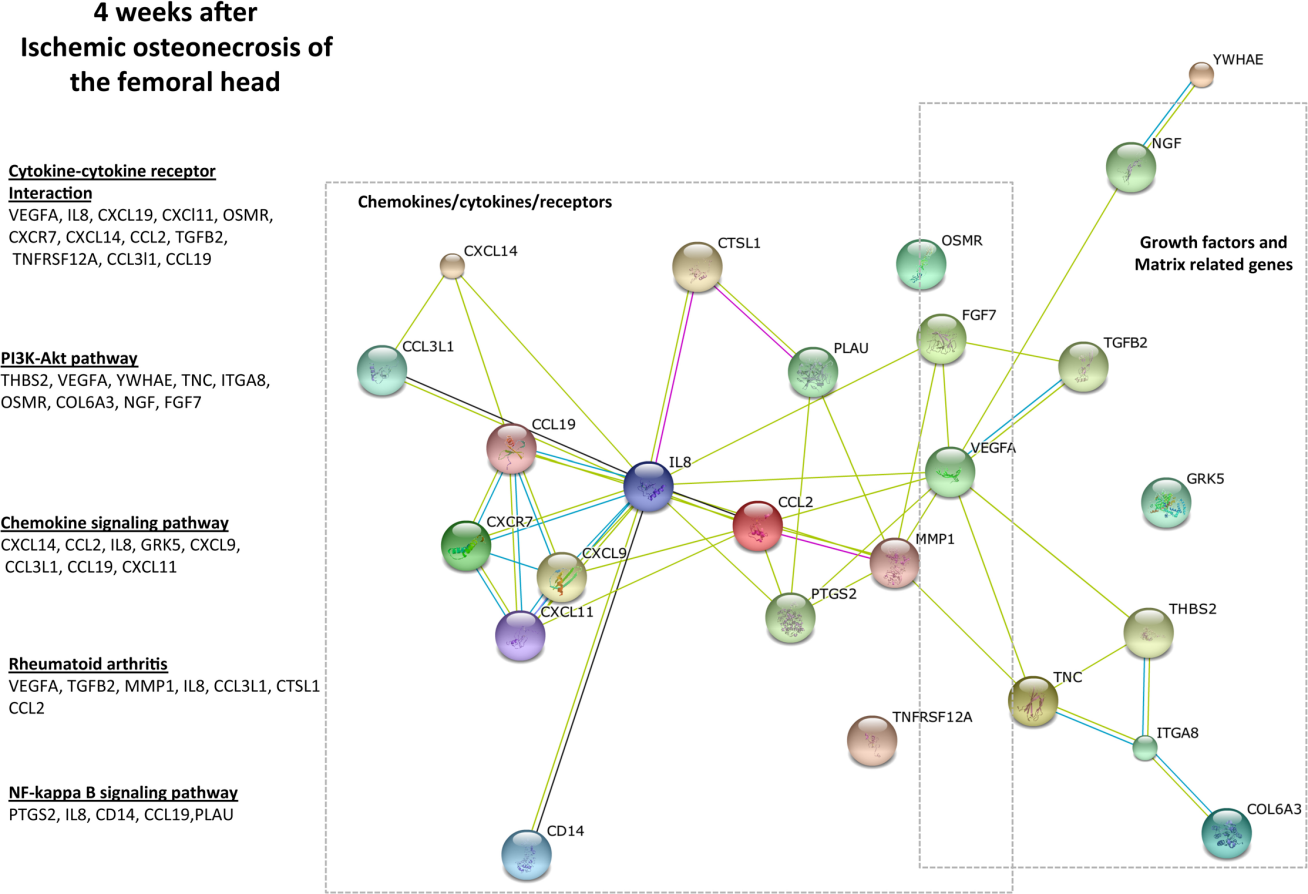
Genes involved in inflammatory response are significantly enriched in the immature articular cartilage at 4 weeks following the induction of ONFH. The genes and the pathways that were significantly enriched (p<0.05, based on t-tests, Bonferroni, FDR) belong to inflammation. The STRING10 network diagram indicates that the different genes are closely related to the inflammatory responses. The clusters of genes indicating chemokines/cytokines/receptors and the relationship to growth factors and matrix related genes show the functional link between matrix and recruitment of inflammatory cells. The nodes indicate individual genes and the connecting lines indicate the relationship. Shorter lines indicate closer relationship. The evidence plot utilizes multiple lines to indicate stronger evidence for the proximity in a functional relationship.

These results from the functional clustering diagram (Fig 3) indicated that genes involved in angiogenesis, inflammation and matrix remodeling were significantly upregulated in the immature articular cartilage at 2 weeks following ONFH.

### At 4 weeks after the induction of ONFH (early repair phase), the genes involved in inflammatory processes were upregulated in the immature articular cartilage

At 4 weeks after ONFH, when there is an early repair process observed in the epiphyseal region of the femoral head [12,14,16,18]. Similar to 24 hours and 2 weeks, response to ischemic injury and angiogenesis were significantly upregulated at 4 weeks after ONFH. But the inflammatory role of immature articular cartilage was predominant at 4 weeks, involving cytokines and chemokines/receptors (Table 3 and S1 Table) that corresponded to the molecular functions involving chemokine/receptor/matrix binding and cytokine activity.

**Table 3:** Biological processes, molecular functions and biological pathways upregulated in the immature articular cartilage at 4 weeks following ONFH.

**DAVID**	**Enrichment**	**Count**	**P value**	**Bonferroni**	**FDR**
**Annotation category**	**score**				
Response to wounding	4.84	21	1.50E-09	2.20E-06	2.40E-06
Inflammatory response	4.84	12	2.70E-05	4.00E-02	4.50E-02
Blood vessel development	3.81	11	3.50E-06	5.30E-03	5.80E-03
**SP_PIR_Keyword**					
Cytokine	4.84	8	9.70E-05	2.30E-02	1.30E+00
Chemotaxis	4.84	5	8.50E-04	1.80E-01	1.10E+00
**GOTERM-MF-FAT**					
Glycosaminoglycan binding	6.42	11	4.70E-08	1.30E-05	6.20E-05
Chemokine activity	3.98	7	7.50E-07	2.10E-04	9.90E-04
Chemokine receptor binding	3.98	7	1.10E-06	3.10E-04	1.50E-03
Cytokine activity	3.98	10	8.80E-06	2.50E-03	1.20E-02
**STRING-PATHWAYS**	**Count**	**P value**	**Bonferroni**	**FDR**	
Cytokine-cytokine receptor interaction	12	3.57E-08	1.02E-05	1.02E-05	
PI3K-Akt signaling pathway	9	1.45E-04	4.17E-02	7.50E-03	
Chemokine signaling pathway	8	9.10E-06	2.61E-03	6.53E-04	
Rheumatoid arthritis	7	6.04E-07	1.73E-04	8.67E-05	
NF-kappa B signaling pathway	5	1.57E-04	4.50E-02	7.50E-03	

**Abbreviations:** ONFH: osteonecrosis of the femoral head; DAVID: The Database for Annotation, Visualization and Integrated Discovery; GOTERM-BP-FAT: gene ontology term-biological process-functional annotation tool; SP_PIR_keyword: SwissPort protein interaction resource keyword, indicates protein categories; GOTERM-MF-FAT: gene ontology term-molecular function-functional annotation tool; STRING-pathways: Search Tool for the Retrieval of Interacting Genes/Proteins; Count: number of genes that are significantly enriched in the indicated annotation category. FDR: false discovery rate. Bonferroni: a statistical test used in the correction for multiple comparisons.

Similar to 24 hours and 2 weeks, the PI3K-Akt signaling pathway (e.g., *VEGFA*, *FN1*) is significantly upregulated at 4 weeks. (Fig 3). Functional clustering revealed gene groups specific to inflammatory mechanisms including chemokine pathways (involving *CCL2*, *CXCL9* among others), cytokine interactions, and importantly genes commonly involved in the inflammatory TNF pathway and the NFkB pathway [27].

These results suggest that the immature articular cartilage plays a significant role in the inflammatory response at 4 weeks following ONFH.

### Assessment of the index genes upregulated in the immature articular cartilage following ONFH

**Fig 4:**
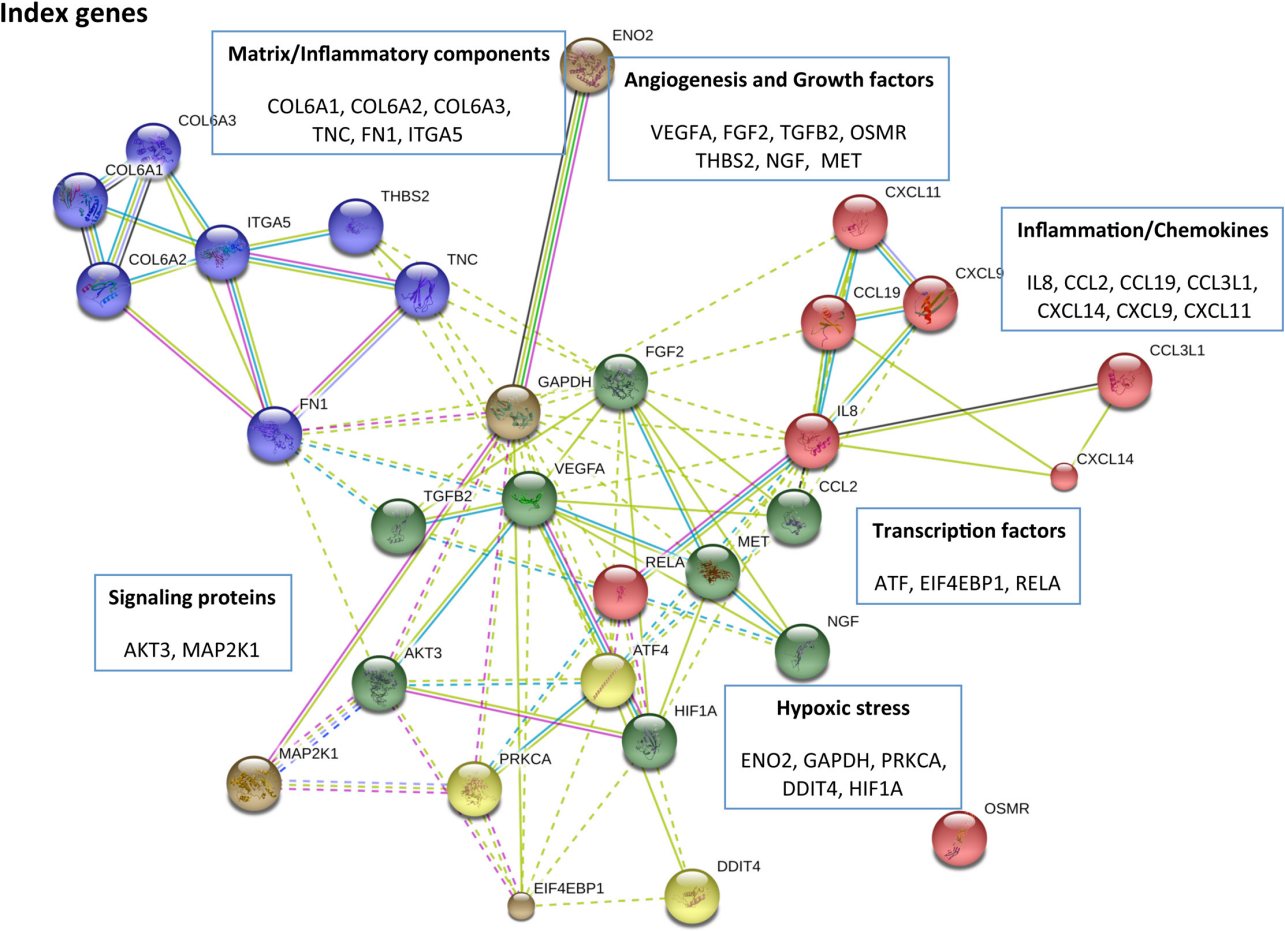
Index genes representing the major roles of the immature articular cartilage. The STRING10 network diagram indicates the functional clustering of the index genes. The nodes indicate individual genes and the connecting lines indicate the relationship. Shorter lines connect the closely related genes. Multiple connectors indicate stronger evidence for the functional relationship.

The analysis of gene lists at 24 hours, 2 weeks and 4 weeks revealed several index genes (genes that were known to be important in biological pathways/disease conditions based on the annotations), suggestive of the major functional roles of the immature articular cartilage. Specifically, growth factors: *VEGFA*, *FGF2*, *TGFB2*, *THBS2*; mediators of inflammation: *IL-6R*, *IL-8*, *CCL2*, *CXCL14*; matrix/remodeling components: *MMP1*, *MMP3*, *CTSL1*, *ITGA5*, *TNC*, *FN*, *Col6A1* and hypoxic response genes: *HIF-1A*, *GAPDH*, *ENO2*, *DDIT* among others were significantly upregulated in the immature articular cartilage following ONFH (Fig 4).

**Fig 5:**
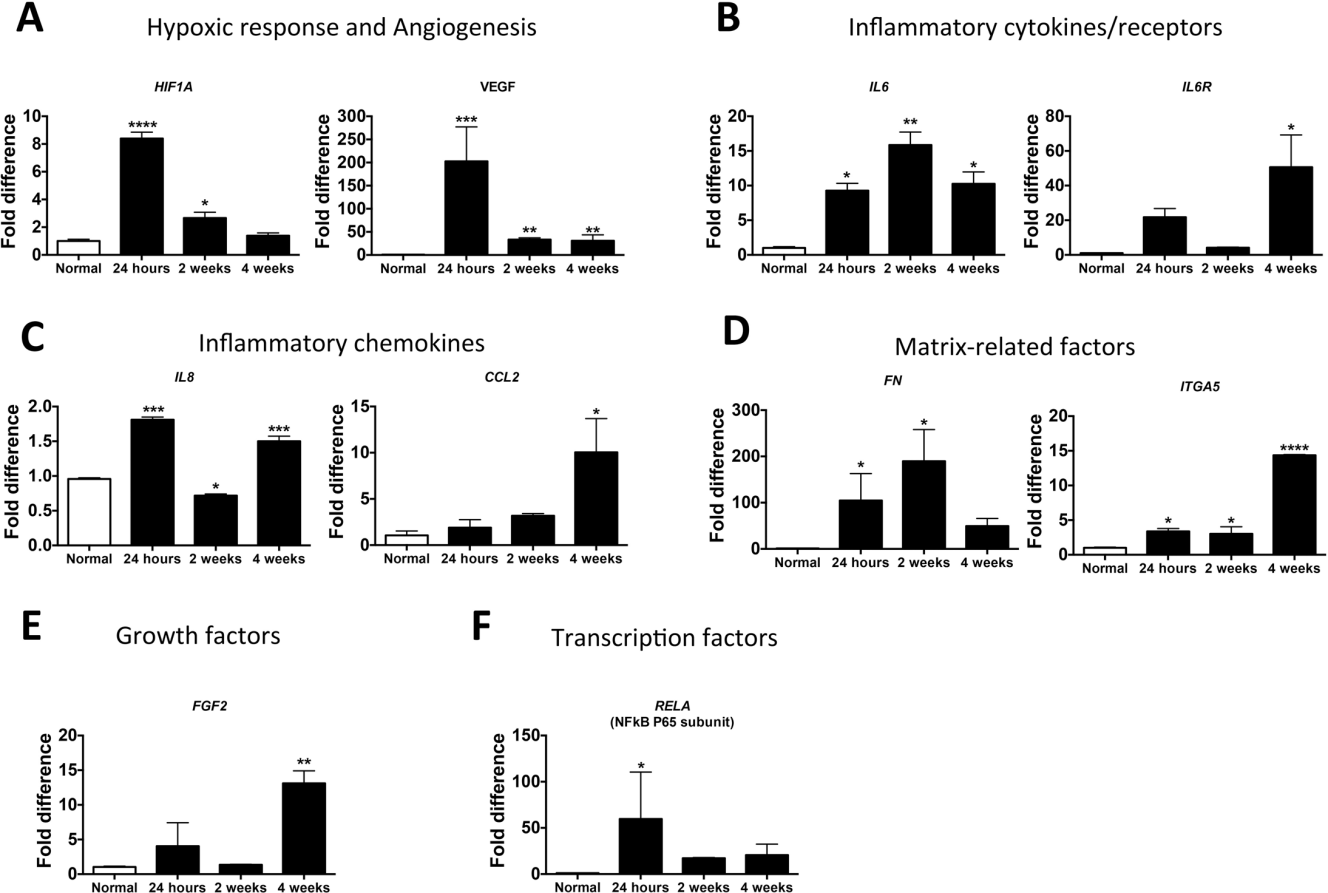
Selected index genes showed increased expression in the immature articular cartilage by qRTPCR analysis. The microarray results were validated by real-time qRTPCR analysis of the expression of selected index genes at 24 hours, 2 weeks and 4 weeks following the induction of ONFH. **(A)** The hypoxic response (*HIFA*, hypoxia inducible factor 1 alpha), angiogenesis (*VEGFA*, vascular endothelial growth factor alpha), **(B)** cytokines/cytokine receptors (*IL6/IL6R*, interleukin 6/interleukin 6 receptor), **(C)** chemokines (*IL8*, interleukin 8, CCL2, chemokine (C-C) motif ligand-2 **(D)** matrix related factors (*FN*, fibronectin; *ITGA5*, integrin alpha v subunit), **(E)** growth factors (*FGF2*, fibroblast growth factor-2) and **(F)** transcription factor (*NFkB p65*, nuclear factor kappa b, subunit p65) were confirmed by qRTPCR. The mRNA levels were normalized to 18sRNA and the data is shown as fold difference relative to normal articular cartilage. A total of n = 4 pigs were used per time point. Statistical analysis was performed using ANOVA and Tukey’s multiple comparison tests. P value * <0.05, **<0.01, ***<0.001, ****<0.0001

The expression levels of genes selected from the key index genes were assessed by a real-time qRTPCR analysis, which validated the increased expression of the genes in the microarray analysis (Fig 5). Index genes that represent hypoxic response (*HIF1A*), angiogenesis (*VEGFA*) (Fig 5A), inflammatory cytokines/receptors/chemokines (*IL6*, *IL6R*, *IL8*, *CCL2*), matrix related factors (*FN*, *ITGA5*) (Fig 5B–5D), growth factors (*FGF2*) (Fig 5E) and inflammatory transcription factors (*RELA*) (Fig 5F) were tested for increased expression. Importantly, similar to previous studies that demonstrated the HIF-1 dependent regulation of the expression of VEGF [19], qRTPCR analysis showed temporal increase in the gene expression of *HIF-1* and *VEGF* in the immature articular cartilage following ischemic osteonecrosis. The significantly increased expression of *IL6*, *IL6R*, *IL8*, *CCL2* and *ITGA5* at 4 weeks also confirmed the involvement of the immature articular cartilage in the inflammatory responses following ONFH.

## Discussion

The major finding of this study is that the immature articular cartilage plays a multi-faceted role following ischemic osteonecrosis of the femoral head (ONFH). Specifically, the genes involved in cellular response to hypoxic stress, angiogenesis, matrix remodeling and inflammation are significantly enriched in the immature articular cartilage following ONFH. The results presented here strongly suggest a temporal adaptation of the transcriptional regulation in the immature articular cartilage from acute ischemic phase to the early repair phase following ischemic osteonecrosis. The main response of the immature articular cartilage at the acute ischemic injury phase (at 24 hours after the induction of ONFH) was the upregulation of the HIF-1A-dependent pathway as a hypoxic stress response, and the upregulation of PI3K-Akt and MAPK signaling pathways that are necessary for cell survival. At the avascular necrosis phase (2 weeks), the genes involved in angiogenesis, inflammation and matrix remodeling were upregulated. At the early repair phase (4 weeks), the genes involved in the inflammatory response through the upregulation of the expression of chemokines, cytokines and the matrix components that are critical in the adhesion of inflammatory cells were upregulated. In all these time points, the PI3K-Akt signaling pathway was consistently involved.

The biological processes and functional pathways presented in this study are consistent with the findings from histologic studies using the piglet model. Previous studies have shown that after the induction of ONFH, the expression of *HIF-1*, *VEGF and SOX9* at the mRNA and protein levels increases from 24 hours up to 2 weeks or 4 weeks [16–19,21]. Histologically, at 2 weeks to 4 weeks following the induction of ONFH, a neovascularization is observed at the periphery of the immature articular cartilage and the infarcted secondary ossification center [17]. A micro-CT assessment following the injection of an intravascular microfil dye showed new blood vessels traversing the immature articular cartilage into the necrotic secondary ossification center [18]. This neovascularization corresponds to increased *HIF1A* and *VEGFA* expression [19]. This process is associated with a vascular tissue invasion of the necrotic marrow space consisting of capillaries, inflammatory and fibroblastic cells. These processes are dependent on angiogenesis (e.g. *VEGFA*), fibrous tissue formation (e.g. *FGF2*, *TGFB2*), matrix remodeling in order to facilitate the invasion of the fibrovascular tissue (e.g. *MMP1*, *MMP3*, *CTSL*) and chemokines involved in the inflammatory cell recruitment (e.g. *IL8*, *CCL2*) [28].

An assessment of the index genes also supports the histological changes previously reported in the immature articular cartilage following ONFH. The upregulation of *HIF1A*, *GAPDH*, *ENO2*, *DDIT* indicate the hypoxic stress response [29] to the induction of ONFH. The upregulation of growth factors *VEGFA*, *FGF2*, *TGFB2*, *THBS2* and matrix/remodeling components *MMP1*, *MMP3*, *CTSL1*, *ITGA5*, *TNC*, *FN*, *Col6A1* indicate the role of articular cartilage in the coupling of angiogenesis and matrix remodeling processes [28,30]. These changes were also accompanied by the increased production of chemokines/ligands *IL8*, *CCL2*, *CXCL9*, *CXCL14* that are involved in the recruitment of inflammatory cells [27, 31]. These findings suggest that the immature articular cartilage plays a complex role in the coupling of angiogenesis, matrix remodeling and inflammation following ONFH.

A major observation in our study is the important role of the immature articular cartilage in inflammation. Several genes involved in inflammation (cytokines/receptors: *IL-6*, *IL-6R*, *TGF-b*, *OSMR*; chemokines *IL8*, *CCL2*, *CXCL9*, *CXCL14*, matrix formation and remodeling during inflammation: *TNC*, *FN1*, *INTGA5*, *Col6A1*, *MMP1*, *MMP3*, *CTSL1*) were upregulated in response to ONFH [22, 27,31].

The role of articular cartilage during inflammatory conditions is also evident in diseases, including osteoarthritis and rheumatoid arthritis [32–35]. In the study by Karlsson et al [32], several genes, including growth factors (*IGF1*, *FGF2*, *LTBP1*, *POSTN*), matrix components (*TNC*, *ASPN*, *VCAN*, *ECM1*, *COL6A2*), and inflammatory components (*IL8*, *CCL2*, *CXCL14*) were upregulated in the diseased cartilage from patients with osteoarthritis compared to the cartilage from normal patients. These changes in gene expression were similar to those observed in the immature articular cartilage following ONFH in our study. These similarities indicate that articular cartilage might perform a multi-functional role during inflammatory conditions, influencing several processes including the matrix formation, remodeling and inflammation. Several genes that were involved in matrix remodeling have also been shown to be upregulated in the damaged regions compared to normal regions of the cartilage obtained from the patients with osteoarthritis [33]. Also, the upregulation of growth factors associated with bone formation, such as *IGF1*, *FGF2*, *LTBP1* and *POSTN* suggest that articular cartilage might also influence bone repair process during these inflammatory conditions. Furthermore, several genes that were annotated in studies on rheumatoid arthritis (*VEGFA*, *TGFB2*, *MMP1*, *MMP3*, *IL8*, *CCL3L1*, *CTSL1*, *CCL2*) [34,35] were also significantly upregulated in the immature articular cartilage in our study.

The porcine articular cartilage is relevant to understanding the inflammatory conditions in the human articular cartilage as suggested in a recent study which showed that the gene expression was similar in the porcine and human chondrocyte micromass cultures [36]. Furthermore, when exposed to TNF-a to mimic inflammatory conditions in osteoarthritis, the genes involved in inflammation (*PTGS2*, *CCL2*, *CXCL14*), ECM components (*Col2A1*, *CILP*, *THBS3*), growth factors (*IGFBP3*, *IGFBP6*) and matrix remodeling enzymes (*MMP1*, *MMP3*, *PDK4*, *HTRA1*) were upregulated similar to our study. In patients with LCPD, a chronic synovial inflammation is associated with a specific and sustained elevation of the pro-inflammatory cytokine interleukin-6 (IL-6) in the synovial fluid [10]. In this study, the immature articular cartilage in piglets showed increased expression of *IL-6* (S1 Table) following ONFH suggesting a possible role in IL-6 elevation.

The upregulation of the genes associated with inflammatory pathways following ONFH suggests that the articular chondrocytes might be exposed to stimuli in the necrotic femoral head that can induce inflammatory responses following an ischemic-hypoxic injury. It is well known that tissue necrosis results in the release of damage associated molecular patterns (DAMPs) [37], which are damaged cell-endogenous and matrix components known to stimulate inflammatory responses. Articular chondrocytes are known to express pattern recognition receptors that recognize the DAMPs [22,38,39], which results in the activation of the inflammatory pathways. An understanding of the molecular mechanisms responsible for the upregulation of genes associated with inflammatory pathways in the immature articular cartilage following ONFH requires further studies.

In conclusion, the immature articular cartilage responds to ONFH by upregulation of genes involved in hypoxic stress response, angiogenesis, matrix remodeling and inflammation. This study provides novel insights into the multi-faceted role of articular cartilage, with inflammation as a key component, in response to ONFH in an immature animal model. The upregulation of several key genes involved in inflammatory response suggest the importance of targeting inflammation in the therapeutic approaches in LCPD.

## Supporting Information

S1 Fig Ischemic osteonecrosis of the femoral head induces a differential gene expression in the immature articular cartilage. (A) At 24 hours, 2 weeks and 4 weeks (n = 4 piglets/time point), RNA was isolated from the normal and osteonecrosis cartilage and was assessed by a microarray analysis. The profile plot shows the changes in expression levels of various Affymetrix probes inferred from the normalized signal intensity across the microarray chip. Maximum changes were observed at 24 hours. This prolife plot demonstrates changes over time, with normal cartilage retaining a similar prolife at all time points. (B) Individual dot plots for each time point were generated by using the Affymetrix Transcriptome Analysis Console (TAC) to indicate the numbers of genes significantly upregulated (>2-fold increase, p<0.05) at each time point.
(TIF)

S1 Table Affymetrix probe ID, Fold increase in osteonecrosis compared to normal, Gene symbols, Gene ID and Ensemble ID, for the differential expressed genes in the immature articular cartilage are shown following ischemic osteonecrosis of the femoral head. (XLS)

S2 Table List of primers and primer sequences used for qRTPCR validation of the upregulation of the index genes in the microarray analysis (DOCX)
