# Supplementary figures and images for: Comprehensive Genome-Wide Transcriptomic Analysis of Immature Articular Cartilage following Ischemic Osteonecrosis of the Femoral Head in Piglets
Source: PLoS One. 2016 Apr 5;11(4):e0153174. doi: 10.1371/journal.pone.0153174 (PMC4821627; doi:10.1371/journal.pone.0153174)

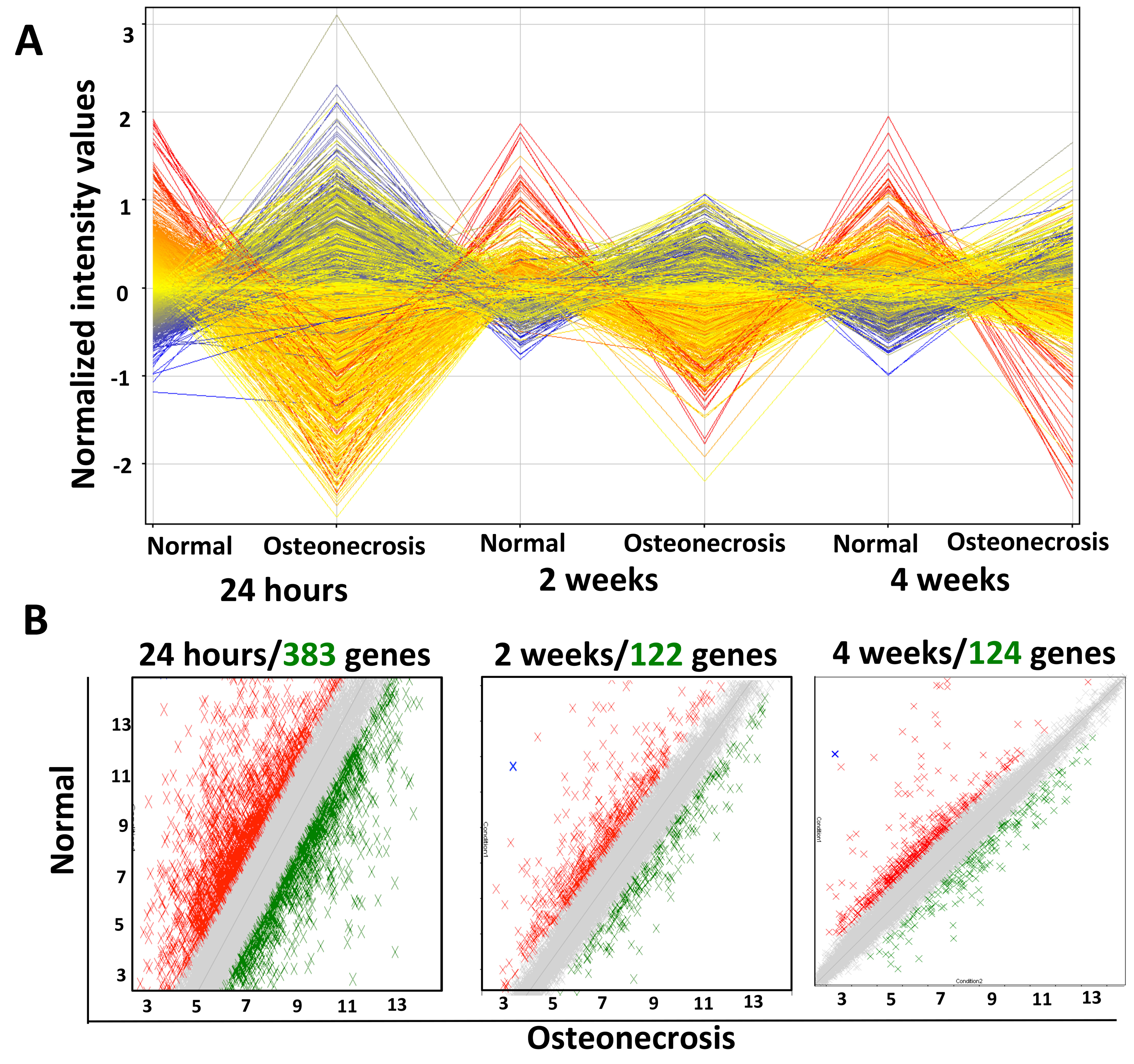

Supplement: S1 Fig — (A) At 24 hours, 2 weeks and 4 weeks (n = 4 piglets/time point), RNA was isolated from the normal and osteonecrosis cartilage and was assessed by a microarray analysis. The profile plot shows the changes in expression levels of various Affymetrix probes inferred from the normalized signal intensity across the microarray chip. Maximum changes were observed at 24 hours. This prolife plot demonstrates changes over time, with normal cartilage retaining a similar prolife at all time points. (B) Individual dot plots for each time point were generated by using the Affymetrix Transcriptome Analysis Console (TAC) to indicate the numbers of genes significantly upregulated (>2-fold increase, p<0.05) at each time point. (TIF) [file pone.0153174.s001.tif]
