# Supplementary material for: Comprehensive Genome-Wide Transcriptomic Analysis of Immature Articular Cartilage following Ischemic Osteonecrosis of the Femoral Head in Piglets
Source: PLoS One. 2016 Apr 5;11(4):e0153174. doi: 10.1371/journal.pone.0153174 (PMC4821627; doi:10.1371/journal.pone.0153174)
Supplement: S2 Table — (DOCX) [file pone.0153174.s003.docx]

**Supplementary table 2.** List of primers and primer sequences used for qRTPCR validation of the upregulation of the index genes in the microarray analysis

| **Porcine gene** | **Forward (5’-3’)** | **Reverse (5’-3’)** |
| --- | --- | --- |
| *HIF1A* | CACACAGAAATGGCCTTGTGA | TCTCCCCCTGCTAGTTAAGGT |
| *VEGF* | GCAAGAAAATCCCTGTGGGC | ACGCGAGTCTGTGTTTTTGC |
| *IL-6* | CCCACCAGGAACGAAAGAGA | TGAAGGCGCTTGTGGAGAG |
| *Il-6R* | GCCGTGTTACTGGTGAGGAA | AACTGGCAGAAAAACCGCTG |
| *IL-8* | TGGGTGCAGAAGAAGGTTGT | AGCCACGGAGAATGGGTTTT |
| *CCL2* | TTGCCCAGCCAGATGCAAT | TCTTCTGTAGCTCATCAGCCG |
| *FN1* | ACGGTATTCAGCTTCCTGGC | GGCGGTGTGGTTCTCCTAAA |
| *ITGA5* | TTGATGCTGATGGACAGGGAT | AAAGCTACCAGGACCACCAAG |
| *FGF2* | AAAGGAGTGTGTGCAAACCG | CGTCTGTAACACATTTAGAAGCCA |
| *RELA* (*NFkBp65*) | GTGAATCGGAACTCGGGGAG | GGCCCGTGAAATACACCTCG |
